# SARS-CoV-2 infection alters mitochondrial and cytoskeletal function in human respiratory epithelial cells mediated by expression of spike protein

**DOI:** 10.1128/mbio.00820-23

**Published:** 2023-07-28

**Authors:** Bonnie H. Yeung-Luk, Gitanjali A. Narayanan, Baishakhi Ghosh, Ara Wally, Esther Lee, Michelle Mokaya, Esha Wankhade, Rachel Zhang, Brianna Lee, Bongsoo Park, Jessica Resnick, Anne Jedlicka, Amanda Dziedzic, Murugappan Ramanathan, Shyam Biswal, Andrew Pekosz, Venkataramana K. Sidhaye

**Affiliations:** 1 Department of Environmental Health and Engineering, Johns Hopkins Bloomberg School of Public Health, Baltimore, Maryland, USA; 2 Department of Medicine, Johns Hopkins School of Medicine, Baltimore, Maryland, USA; 3 Department of Biochemistry and Molecular Biology, Johns Hopkins Bloomberg School of Public Health, Baltimore, Maryland, USA; 4 Epigenetics and Stem Cell Aging, National Institute of Aging, National Institutes of Health, Baltimore, Maryland, USA; 5 W. Harry Feinstone Department of Microbiology and Immunology, Johns Hopkins Bloomberg School of Public Health, Baltimore, Maryland, USA; 6 Department of Otolaryngology-Head and Neck Surgery, Johns Hopkins Outpatient Center, Baltimore, Maryland, USA; The Ohio State University School of Medicine, Columbus, Ohio, USA; Dhamar University, Irbid, Yemen

**Keywords:** COVID-19, SARS-CoV-2, lung epithelia, bioenergetics, cytoskeleton, actin

## Abstract

Coronavirus disease 2019 (COVID-19) is caused by severe acute respiratory syndrome coronavirus 2 (SARS-CoV-2, SCV2), which has resulted in higher morbidity and mortality rate than other respiratory viral infections, such as Influenza A virus (IAV) infection. Investigating the molecular mechanisms of SCV2-host infection vs IAV is vital in exploring antiviral drug targets against SCV2. We assessed differential gene expression in human nasal cells upon SCV2 or IAV infection using RNA sequencing. Compared to IAV, we observed alterations in both metabolic and cytoskeletal pathways suggestive of epithelial remodeling in the SCV2-infected cells, reminiscent of pathways activated as a response to chronic injury. We found that spike protein interaction with the epithelium was sufficient to instigate these epithelial responses using a SCV2 spike pseudovirus. Specifically, we found downregulation of the mitochondrial markers SIRT3 and TOMM22. Moreover, SCV2 spike infection increased extracellular acidification and decreased oxygen consumption rate in the epithelium. In addition, we observed cytoskeletal rearrangements with a reduction in the actin-severing protein cofilin-1 and an increase in polymerized actin, indicating epithelial cytoskeletal rearrangements. This study revealed distinct epithelial responses to SCV2 infection, with early mitochondrial dysfunction in the host cells and evidence of cytoskeletal remodeling that could contribute to the worsened outcome in COVID-19 patients compared to IAV patients. These changes in cell structure and energetics could contribute to cellular resilience early during infection, allowing for prolonged cell survival and potentially paving the way for more chronic symptoms.

**IMPORTANCE:**

COVID-19 has caused a global pandemic affecting millions of people worldwide, resulting in a higher mortality rate and concerns of more persistent symptoms compared to influenza A. To study this, we compare lung epithelial responses to both viruses. Interestingly, we found that in response to SARS-CoV-2 infection, the cellular energetics changed and there were cell structural rearrangements. These changes in cell structure could lead to prolonged epithelial cell survival, even in the face of not working well, potentially contributing to the development of chronic symptoms. In summary, these findings represent strategies utilized by the cell to survive the infection but result in a fundamental shift in the epithelial phenotype, with potential long-term consequences, which could set the stage for the development of chronic lung disease or long COVID-19.

## INTRODUCTION

Infection with coronavirus (SARS-CoV-2, SCV2) causes coronavirus disease 19 (COVID-19) that has spread throughout the world. COVID-19 leads to a high incidence of pneumonia, acute respiratory distress syndrome, and death (1, 2). SCV2 entry into host cells requires binding between the angiotensin-converting enzyme-2 (ACE2) receptor and the viral receptor-binding domain of the spike protein. SCV2 entry can occur by one of two paths depending on the variant. Entry at the plasma membrane occurs through the cleavage of the spike protein by serine protease transmembrane serine protease 2 (TMPRSS2) or related serine proteases (3, 4) followed by the cells via endocytosis. Alternatively, virus particles can undergo early endocytosis with the spike protein proteolytically activated by cathepsin-like proteases in endosomes. By comparison, the seasonal influenza virus (influenza A and B virus, IAV, and IBV) enters the host cells via the binding of hemagglutinin to sialic acid residues, followed by internalization and low pH mediated triggering of hemagglutinin-mediated membrane fusion to allow for the transfer of viral ribonucleoproteins to the host cytoplasm (5, 6). Despite these distinct mechanisms of cell entry and viral spread, both viruses infect similar cells in the lung epithelium. However, the mortality rate of COVID-19 is substantially higher than influenza (7). Additionally, there is growing evidence for the increasing prevalence of “long COVID” and potentially persistent radiographic lung changes (8) raising the question of divergent long-term cellular responses (9). Therefore, we studied the epithelial responses to the SCV2 and IAV infections, including host immune response, tissue injury, and cellular metabolism (10 - 12).

Airway epithelium, the first point of contact for both IAV and SCV2, protects the lung against infection by maintaining a physical barrier impermeable to access to subepithelial tissues and an immunologic barrier triggering antiviral immune responses (13, 14). Indeed, as both upper and lower airway epithelial cells are infected by both viruses, comparing IAV and SCV2 in both these epithelial compartments provides a more thorough understanding of the cellular responses triggered by these viruses, with the goal of identifying critical pathways that distinguish the effects of the two viruses and could explain the altered clinical presentations. In this study, we assessed differential gene expression of SCV2 vs IAV infection in human nasal epithelial cells (hNECs) to elucidate any distinct cellular responses between the two viruses that may lead to differences in clinical presentation. We found significantly less cytotoxicity and activation of cell death pathways in the SCV2-infected epithelia. However, the surviving epithelia demonstrated alterations in both cytoskeletal and metabolic pathways suggestive of epithelial remodeling and plasticity in the SCV2-infected cells, reminiscent of pathways activated in chronic injury responses. These strategies provide cellular resilience but could pave the way for more chronic symptoms by changing epithelial structure and function. Our study provides evidence of distinct cellular responses to SCV2 and IAV infection that could result in these divergent clinical outcomes and raises the possibility that the cell survival and resilience that were observed with SCV2 permits the development of long covid.

## RESULTS

### Transcriptome analysis of human nasal cells with IAV and SCV2 infections

**Fig 1:**
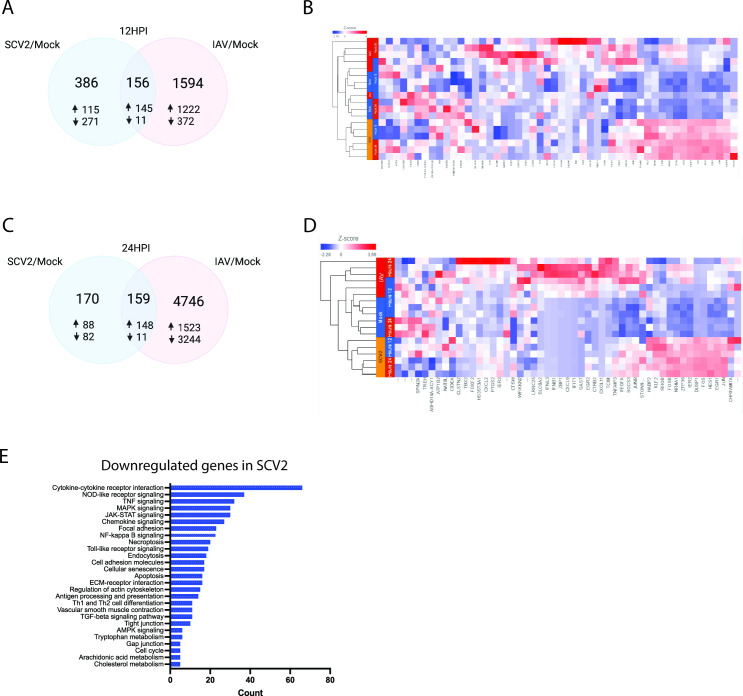
Transcriptomic profiling of human nasal cells with influenza A and SARS-CoV-2 infection. A (IAV) and SARS-CoV-2 (SCV2) infection. Venn diagrams (A and C) and heatmap (B and D) of differentially expressed genes in IAV and SCV2 vs mock at 12 hpi (A and B) and 24 hpi (C and D). Hierarchical clustering was performed to generate the heat maps. The color scale in the heatmaps corresponds to *z*-scores (standardized expression values). (**E**) The KEGG pathway enrichment analysis of the downregulated genes in SCV2 vs IAV at 24 hpi.

**Fig 2:**
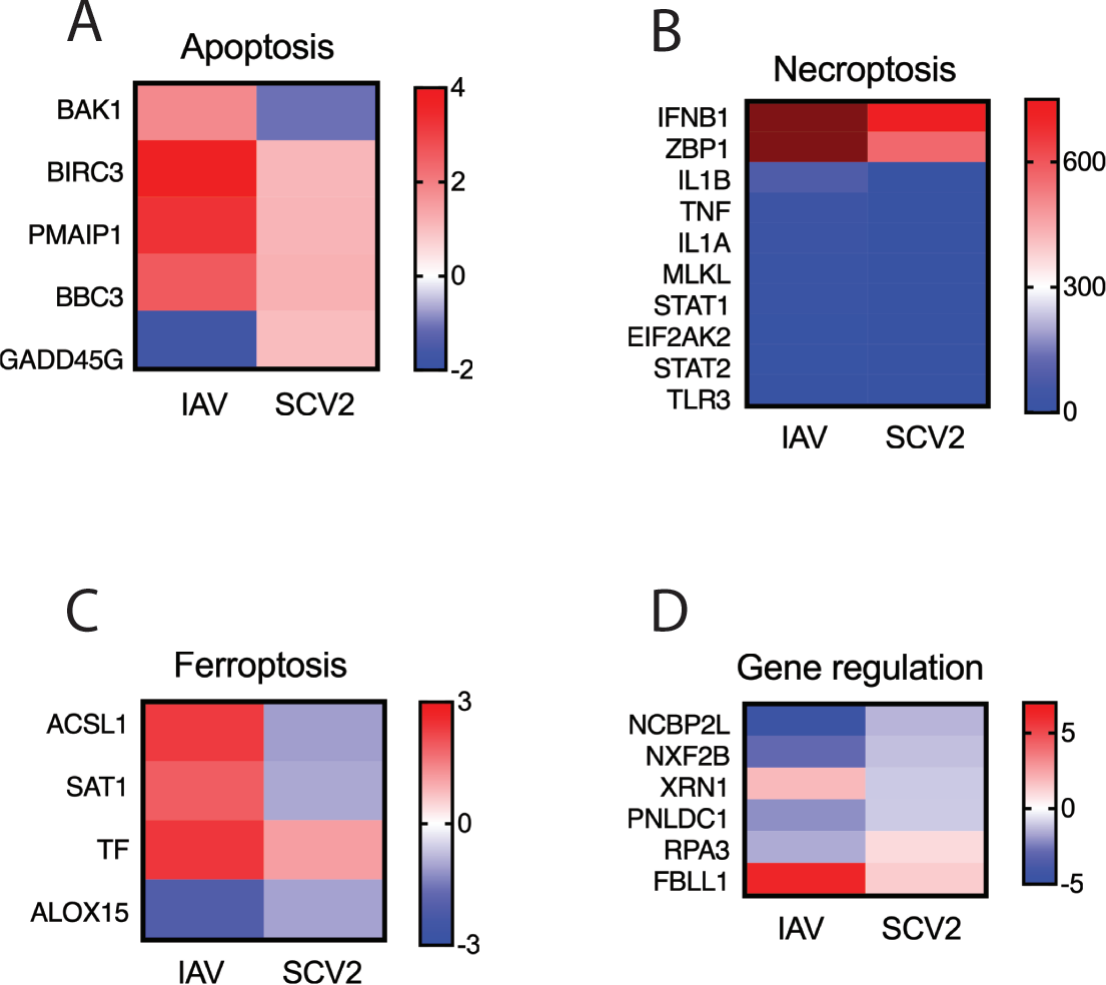
SCV2 decreases cell death signaling and promotes gene regulation. Using KEGG pathway analysis, differential expression of genes involved in (**A**) apoptosis, (**B**) necroptosis, (**C**) ferroptosis, and (**D**) gene regulation was shown in IAV/mock and SCV2/mock at 24 hpi.

Nasal cells are the first site of viral contact harboring high levels of ACE2 and TMPRSS2 (15 - 17). Therefore, hNECs at the air-liquid interface (ALI) were infected with IAV and SCV2 for 12 and 24 h post infection (hpi) (MOI 1), and differentially expressed genes (DEGs) were identified. Eighteen RNA-seq libraries (three experimental replicates for each treatment and timepoint, including mock-infected controls) were prepared. We have previously confirmed that fully differentiated hNECs at ALI strongly correlate with cells freshly isolated from nasal brushes (18). We obtained 38,735,845 to 173,488,265 raw reads for each sequencing samples. After Partek Flow gene-specific analysis with comparisons (cutoff 1.5-fold changes with *P* < 0.05), 386 DEGs (271 downregulated, 115 upregulated), and 1,594 DEGs (372 downregulated, 1,222 upregulated) were observed in SCV2/Mock and IAV/Mock at 12 hpi, respectively, (Fig. 1A) as shown in the heatmap (Fig. 1B). At 24 hpi, there were 170 DEGs (82 downregulated, 88 upregulated) in SCV2/mock and 4746 DEGs (3,244 downregulated, 1,523 upregulated) in IAV/mock (Fig. 1C) as shown in the heatmap (Fig. 1D). As gene expressions were differentially expressed between SCV2 and IAV at 24 hpi, pathway analysis was performed using KEGG Mapper (https://www.genome.jp/kegg/mapper/). Compared to IAV, inflammation, signal transduction, cell adhesion, cell death, and metabolism were enriched in the downregulated genes in SCV2, but no enriched pathway was found in the upregulated genes in SCV2 (Fig. 1C). As COVID-19 patients exhibit lower cytokine levels than patients with influenza (19), in this study, we focused on the early changes in metabolism and cell adhesion pathways in SCV2 and IAV infection. In general, we found the suppression of cell death pathways with SCV2 infection, with decreased apoptosis (Fig. 2A), necroptosis (Fig. 2B), and ferroptosis (Fig. 2C) in SCV2. Of note, general regulation of cellular transcription remained upregulated with SCV2 infection compared to IAV (Fig. 2D). These data indicate that the epithelium is more likely to survive SCV2 infection than IAV at this timepoint.

**Fig 3:**
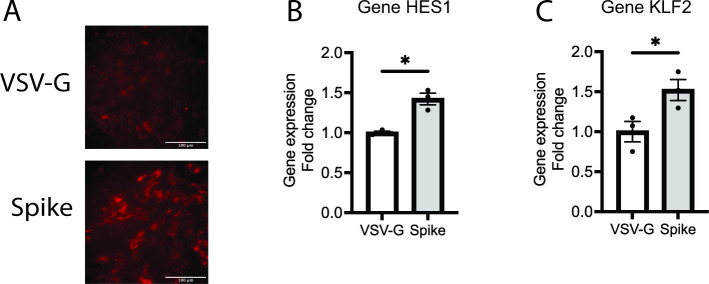
SCV2 pseudovirus induces SCV2-specified gene expression in NHBE. NHBE cells were infected with SCV2 spike or control pseudovirus for 72 h, and (**A**) the infection efficiency was imaged by DsRed fluorescent signal (32 ×, scale bars of 100 µm). Gene expression of (**B**) HES1 and (**C**) KLF2 was examined by qPCR and normalized by housekeeping gene GAPDH. The error bars represent ±standard error of the mean (SEM) (*n* = 3). Statistics were determined by Welch’s test, with *P* < 0.05 considered statistically significant. GAPDH, glyceraldehyde-3-phosphate dehydrogenase; HES1, hes family BHLH transcription factor 1; KLF2, KLF transcription factor 2; NHBE, normal human bronchial epithelial; Spike, SARS-CoV-2 spike glycoprotein; VSV-G, VSV glycoprotein.

We found three genes that were commonly activated with infection with SCV2 at both 12 and 24 hpi (DUSP1, HES1, KLF2) (Table 1) and some non-congruent genes at 12 hpi (CCL5, ZBP1, NRXN2, STAB1, SLC25A47, CXCL11) and 24 hpi (FOXA2, RHOB) (Table 2). To assess the dependency on interaction with the spike protein, we infected normal human bronchial epithelial (NHBE) cells at the air-liquid interface with SCV2 spike pseudovirus. The vesicular stomatitis virus (VSV-G) pseudovirus is an enveloped negative-stranded RNA virus (20, 21). Engineering the SCV2 spike protein into the VSV-G fused fluorescent protein DsRed allows us to analyze virus entry and determine the specific downstream mechanisms induced by the spike protein. Acquiring the pseudovirus model by infection of SCV2 spike pseudovirus (Spike) or control virus (VSV-G) to NHBE at ALI for 72 h, the infection efficiency was examined by red fluorescent protein (DsRed)-tagged pVSV-G (Fig. 3A), which was calculated to be approximately 80%–90% of ciliated epithelial cells. SCV2 Spike pseudovirus infection significantly induced gene expression of HES1 (1.42-fold in Spike vs VSV-G, *P* = 0.0232) (Fig. 3B) and KLF2 (1.52-fold in Spike vs VSV-G, *P* = 0.0466) (Fig. 3C) but not significant in DUSP1 (increased by 1.15-fold in Spike vs VSV-G, *P* = 0.1865) (data not shown).

**TABLE 1:** List of SCV2-specific genes expressed in both 12 and 24 hpi

		12 hpi		24 hpi	
Gene ID	Gene name^*a*^	Fold change (SCV/Mock)	*P-*value	Fold change (SCV2/Mock)	*P-*value
ENSG00000120129	DUSP1	3.92E + 00	6.65E− 10	4.48E + 00	1.84E − 10
ENSG00000114315	HES1	1.56E + 00	2.63E − 06	1.66E + 00	5.72E − 07
ENSG00000127528	KLF2	2.87E + 00	6.85E − 03	3.04E + 00	5.26E − 03

^*a*^ DUSP1, dual specificity phosphatase 1; HES1, hes family bHLH transcription factor 1; KLF2, Kruppel-like factor 2.

**TABLE 2:** List of non-congruent genes at 12 and 24 hpi

		Fold change	Fold change	*P-*value	Fold change	*P-*value
Gene ID	Gene name^*a*^	(SCV2-IAV)	(SCV2/Mock)	(SCV2/Mock)	(IAV/Mock)	(IAV/Mock)
12 hpi						
ENSG00000271503	CCL5	−6.10E + 01	−5.37E + 00	2.64E − 02	5.57E + 01	5.83E − 03
ENSG00000124256	ZBP1	−7.00E + 01	−2.22E + 00	3.71E − 02	6.78E + 01	4.65E − 05
ENSG00000110076	NRXN2	−1.91E + 02	−1.84E + 02	1.50E − 02	7.20E + 00	2.53E − 02
ENSG00000010327	STAB1	−2.12E + 02	−1.88E + 02	2.70E − 02	2.45E + 01	8.25E − 03
ENSG00000140107	SLC25A47	−2.33E + 02	−1.84E + 02	1.49E − 02	4.97E + 01	1.61E − 03
ENSG00000169248	CXCL11	−7.55E + 02	−5.43E + 02	1.29E − 02	2.12E + 02	4.28E − 04
24 hpi						
ENSG00000125798	FOXA2	1.73E + 02	8.09E + 00	1.91E − 02	−1.65E + 02	1.26E − 02
ENSG00000143878	RHOB	2.81E + 00	1.58E + 00	2.12E − 04	−1.23E + 00	3.24E − 02

^*a*^ CCL5, C-C Motif Chemokine Ligand 5; CXCL11, CXC motif chemokine ligand 11; FOXA2, Forkhead Box A2; NRXN2, Neurexin 2; RHOB, ras homolog family member B; STAB1; SLC25A47, solute carrier family 25 member 47; Stabilin-1; ZBP1, Z-DNA-binding protein.

### SCV2 alters metabolism in the host cells

**Fig 4:**
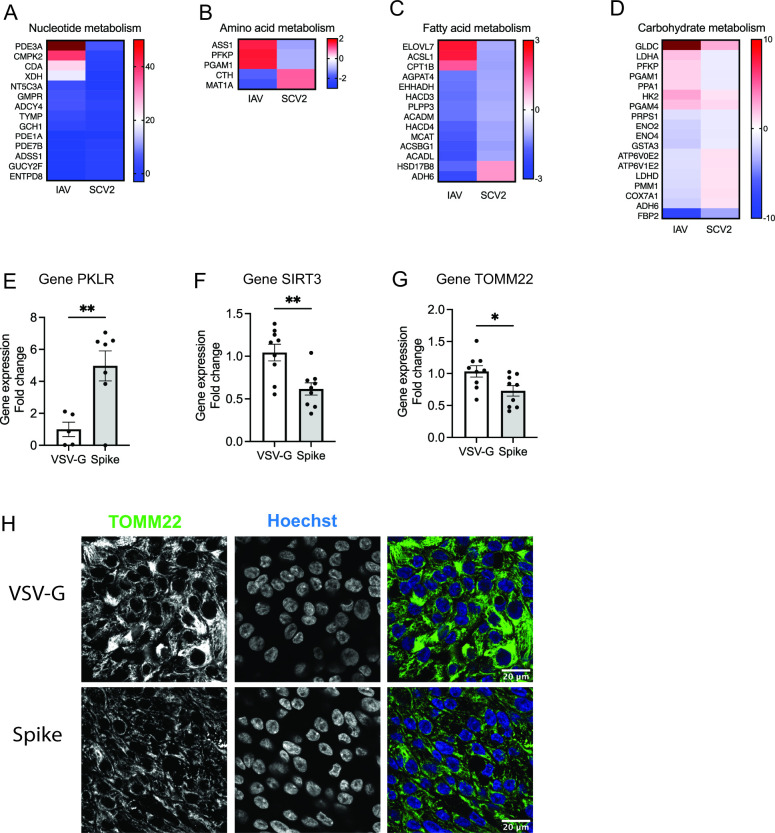
SCV2 infection enhances metabolism in the host cells. Using KEGG pathway analysis, differentially expressed genes involved in (**A**) nucleotide, (**B**) amino acid, (**C**) fatty acid, and carbohydrate metabolisms were shown in IAV/mock and SCV2/mock at 24 hpi. After 72 h of pseudovirus infection in NHBE cells, gene expression of (**E**) glycolysis rate limiting PKLR, (**F**) SIRT3, and (**G**) mitochondrial marker TOMM22 was measured by qPCR and normalized by housekeeping gene GAPDH (5–9 inserts from two donors), and (**H**) the mitochondrial localization was observed by TOMM22 immunofluorescent staining. The immunofluorescence images were taken at 63× oil objective, scale bars of 20 µm. The error bars represent ±standard error of the mean (SEM) (two donors). Statistics were determined by Welch’s test, with *P* < 0.05 considered statistically significant. GAPDH: glyceraldehyde-3-phosphate dehydrogenase. PKLR, pyruvate kinase L/R; SIRT3, NAD+-dependent protein deacetylases 3; TOMM22, translocase of the outer membrane of mitochondria; Spike, SARS-CoV-2 spike glycoprotein; VSV-G, VSV glycoprotein.

**Fig 5:**
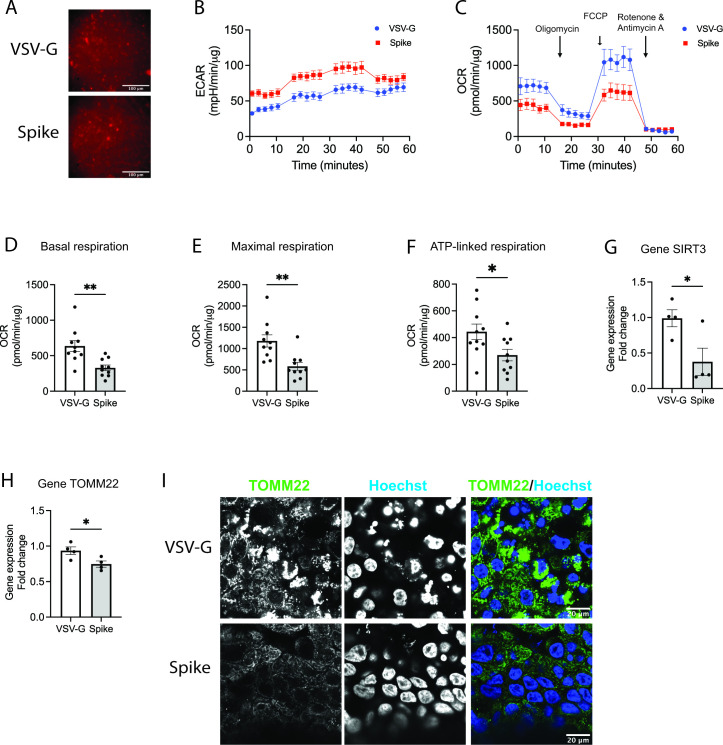
SCV2 spike protein infection impairs metabolism in human epithelial cells. (**A**) The pseudovirus infection efficiency of Calu3 cells after 48 h post infection was examined by DsRed fluorescent signal (32×, scale bars of 100 µm). (**B**) ECAR and (**C**) OCR were examined in the pseudovirus-infected Calu3 cells. (**D**) Basal respiration, (**E**) maximal respiration, and (**F**) ATP-linked respiration were shown. Gene expression of (**G**) SIRT3 and (**H**) TOMM22 as well as (**I**) immunofluorescence of TOMM22 (green) and Hoechst (blue) staining were taken at 63× oil objective, scale bar of 20 µm. The error bars represent ±standard error of the mean (SEM) (4–10 inserts/group). Statistics were determined by Welch’s test, with *P* < 0.05 considered statistically significant. ECAR, extracellular acidification rate; OCR, oxygen consumption rate; RHOB, ras homolog family member B; SIRT3, NAD+-dependent protein deacetylases 3; TOMM22, translocase of the outer membrane of mitochondria; Spike, SARS-CoV-2 spike glycoprotein; VSV-G, VSV glycoprotein.

Using KEGG Mapper, DEGs in SCV2 infection increased metabolisms in the host cells including nucleotide (Fig. 4A), amino acid (Fig. 4B), fatty acid (Fig. 4C), and carbohydrate metabolisms (Fig. 4D). Gene expression of pyruvate kinase L/R, PKLR, encoded the protein that is critical in glycolysis, was upregulated in the spike-infected NHBE cells at 72 h post infection (4.71-fold in Spike vs VSV-G, *P* = 0.0047) (Fig. 4E). The Spike infection decreased NAD+-dependent protein deacetylases 3, SIRT3, that maintains mitochondrial metabolic activity (22) (0.59-fold in Spike vs VSV-G, *P* = 0.0031) (Fig. 4F) and translocase of the outer membrane of mitochondria, TOMM22, gene expression (0.73-fold in Spike vs VSV-G, *P* = 0.0229) (Fig. 4G). The corresponding decrease in TOMM22 protein expression in Spike-infected NHBE cells was confirmed by immunofluorescence (Fig. 4H). Next, we utilized a differentiated human non-small-cell lung cancer cell line Calu3 at ALI, as this cell line has been demonstrated to be highly permissive to SCV2 with similar characteristics as NHBE with high barrier integrity (23 - 27). Differentiated Calu3 cells were infected with the Spike or control virus and the infection efficiency was examined by immunofluorescence of DsRed-tagged pVSV-G and DsRed-tagged pVSV-S at 48 h post infection (Fig. 5A). They both had equivalent infection efficiency of approximately 70%. Extracellular acidification rate (ECAR) and oxygen consumption rate (OCR) were measured through seahorse analysis in the pseudovirus-infected Calu3 cells. ECAR is a measure of lactate production. The Spike-infected Calu3 cells exhibited an increase in ECAR (Fig. 5B) and a decrease in OCR compared to the control virus-infected cells (Fig. 5C), which has consisted with the finding that the increased PKLR gene expression in the spike-infected NHBE cells (Fig. 4E). Of note, the ECAR and OCR of the control virus-infected cells were consistent with uninfected Calu3 cells (28). Specifically, the Spike infection significantly decreased basal respiration (0.52-fold in Spike vs VSV-G, *P* = 0.0034) (Fig. 5D), which was similar to the effect of SCV2 infection in Calu3 cells as compared to the uninfected cells (29). Particularly, maximal respiration (0.5-fold in Spike vs VSV-G, *P* = 0.0032) (Fig. 5E) and ATP-linked respiration (0.61-fold in Spike vs VSV-G, *P* = 0.027) (Fig. 5F) were decreased in the Spike infection as compared to VSV-G. Furthermore, we verified similar decreases in gene expression of SIRT3 (0.38-fold in Spike vs VSV-G, *P* = 0.0418) (Fig. 5G) and TOMM22 (0.80-fold in Spike vs VSV-G, *P* = 0.0366) (Fig. 5H) as well as immunofluorescence signal intensity of TOMM22 (Fig. 5I) in the Spike-infected Calu3 cells. The finding suggests that SCV2 infection dysregulates mitochondrial function.

### SCV2 alters epithelium persistently for chronic inflammatory changes

**Fig 6:**
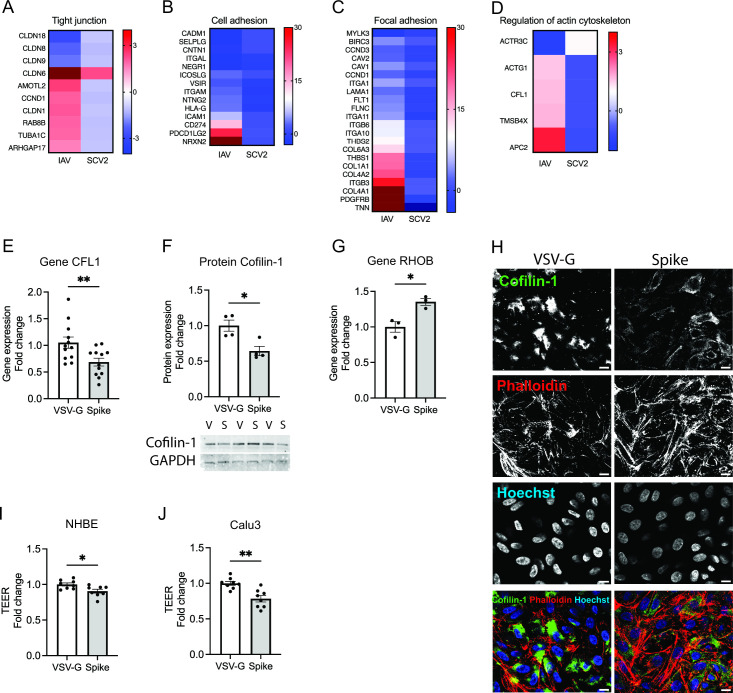
SCV2 infection disrupts cell-cell adhesion in the human epithelium. Using KEGG pathway analysis, differential expression of genes involved in (**A**) tight junction, (**B**) cell adhesion, (**C**) focal adhesion, and (**D**) regulation of actin cytoskeleton was shown in IAV/mock and SCV2/mock at 24 hpi. After 72 h of the pseudovirus infection in NHBE cells, expressions of (**E**) CFL1 transcript, (**F**) CFL1 protein, and (**G**) RHOB transcript were examined (*n* = 3–12). (**G**) Immunofluorescence of TOMM22 (green) and Hoechst (blue) staining was taken at 63× oil objective, scale bar of 10 µm. TEER was measured in the pseudovirus-infected (**H**) NHBE cells and (**I**) Calu3 cells (*n* = 8). The error bars represent ±standard error of the mean (SEM). Statistics were determined by Welch’s test, with *P* < 0.05 considered statistically significant. CFL1, cofilin-1; ECAR, extracellular acidification rate; GAPDH, glyceraldehyde-3-phosphate dehydrogenase; OCR, oxygen consumption rate; TEER, transepithelial electrical resistance; TOMM22, translocase of the outer membrane of mitochondria; Spike, SARS-CoV-2 spike glycoprotein; VSV-G, VSV glycoprotein.

**Fig 7:**
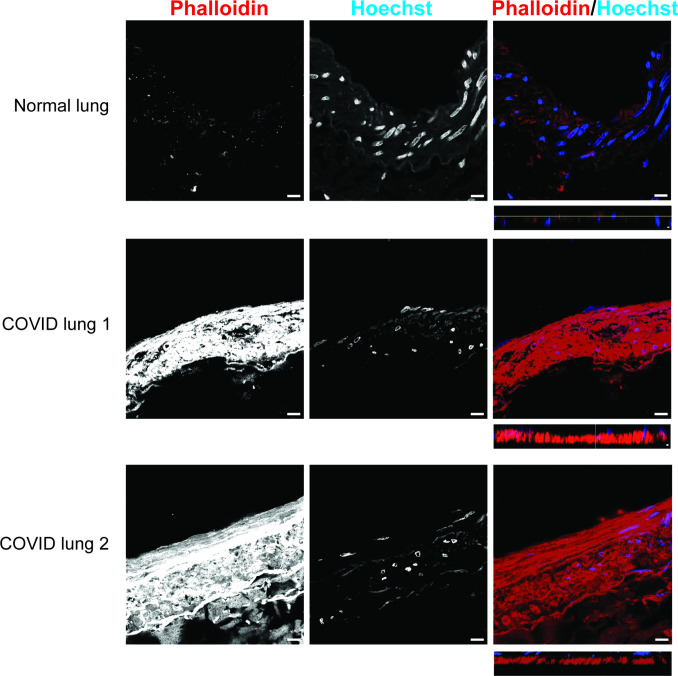
F-actin is increased in human lungs with COVID-19. Immunofluorescent staining of phalloidin (red) and Hoechst (blue) was performed in human lungs from normal and COVID-19 patients. The immunofluorescence images were taken at 63× oil objective (scale bars of 10 µm).

Airway epithelium is a frontline of defense against pathogen. Epithelial permeability is maintained by the apical junctional complexes that include tight junctions and adhesion junction and link with cellular cytoskeleton (30). In addition, focal adhesion and cytoskeleton encompass a network to maintain the proper positioning of a cell’s shape and positioning of its constituent organelles (31). SCV2-infected hNECs generally decreased genes involved in the tight junction (Fig. 6A), cell adhesion (Fig. 6B), focal adhesion (Fig. 6C), as well as regulation of actin cytoskeleton (Fig. 6D). Notably, loss of cofilin-1, an actin-severing protein, occurs in chronically injured epithelium, as seen with recurrent insults that result in chronic obstructive pulmonary disease (COPD) (32). We have found that gene expression of cofilin-1, CFL1, was downregulated in both SCV2-infected hNECs (Fig. 6D) and the spike-infected NHBE cells (0.65-fold in Spike vs VSV-G, *P* = 0.0099) (Fig. 6E). The spike infection in NHBE cells caused the loss of cofilin-1 protein (0.65-fold in Spike vs VSV-G, *P* = 0.0134) (Fig. 6F). In our RNA sequencing data, we also observed that Ras homolog family member B (RHOB), which plays a vital role in cell-cell actin-based adherens junctions (33), was reversibly expressed between SCV2 and IAV2-infected hNECs (increased in SCV2 but decreased in IAV infections) (Table 2). In the pseudovirus-infected NHBE cells, we found RHOB was induced in Spike by 1.35-fold in spike vs VSV-G (*P* = 0.0216) (Fig. 6G). As expected with a decrease in actin-severing, the localization of the decreased cofilin-1 and the increased F-actin (stained by phalloidin) was observed in pseudovirus-infected cells by immunofluorescence (Fig. 6H). Of note, the loss of cofilin-1 was associated with the decreased barrier integrity in NHBE (0.91-fold in Spike vs VSV-G, *P* = 0.0179) (Fig. 6I) and in Calu3 (0.78-fold in Spike vs VSV-G, *P* = 0.0015) (Fig. 6J). To confirm the increased F-actin in SCV2 infection, the immunofluorescent intensity of phalloidin was significantly higher in the lungs of COVID-19 patients compared to healthy individuals (Fig. 7) with *xz*-images showing the increase in phalloidin throughout the thickness of the tissue slice. The data imply that SCV2 infection dysregulates epithelial barrier integrity through the loss of cofilin-1 and increased F-actin.

## DISCUSSION

Though respiratory viruses all initially engage with airway epithelial cells, the mechanisms of the initial immune response to infection are distinct and can result in divergent clinical responses (34, 35). Moreover, there is increasing recognition that SARS-CoV-2 infection can lead to prolonged symptoms or “long-COVID” and may even have persistent radiographic changes but the biological basis of which remains unknown. Here, we compared the transcriptomic profile of the SCV2 vs IAV infection of the nasal epithelium to identify distinct viral responses. Surprisingly, we found that IAV resulted in much more injury to the epithelium at 24 h after infection compared to SCV2. In fact, the early epithelial responses to SCV2 were quite mild and very similar to that of the mock control. By 24 h after the IAV infection, cell death pathways were activated, which did not occur with the SCV2 infection. In contrast, we saw evidence of altered cell structure and metabolism, suggestive of cellular remodeling in response to the SCV2 infection. Infection with SCV2, moreover, caused a downregulation in the protein cofilin-1, and impaired cell-cell adhesion, a finding that is recapitulated with infection with the spike protein.

Although we did not focus on the inflammatory response, we did see an early upregulation of epithelial inflammatory processes. In both 12 and 24 hpi, we found three genes were specifically upregulated in SCV2-infected hNECs (DUSP1, HES1, KLF2). Dual specificity phosphatase (DUSP1) is a negative regulator of the p38 MAPK signaling and diminishes the inflammatory response (36, 37). DUSP1 has been shown to be downregulated at 24 h SCV2-infected human epithelial lung cell line A549 (MOI 0.2) (38), but we found early upregulation in the primary cells. HES, hes family BHLH transcription factor 1 (HES1), a basic helix-loop-helix transcriptional repressor, is a target gene of Notch signaling (39). As notch signaling positively regulates protease furin, protease ADAM Metallopeptidase Domain 17 (ADAM17), and angiotensin-converting enzyme 2 (ACE2) to promote SCV2 entry and infection to the host cells (40). Krüppel-like factor 2 (KLF2) is a transcriptional factor that suppresses inflammatory responses (41). Although we have found upregulation of KLF2 in the nasal at these early timepoints, the study of lung autopsies suggests downregulation in KLF2 in later stages of the disease in patients who succumbed to COVID-19 (42). The role of KLF2 in early vs later disease can be the focus of future studies.

Interestingly, we found the Ras homolog family member B (RHOB), a gene that belongs to the Rho GTPase family, was not congruently expressed between SCV2- and IAV infections at 24 hpi (Table 2). RHOB plays a vital role in cell-cell actin-based adherens junctions through ROCK/LIMK1 signaling pathway (33), a pathway highly implicated in actin cytoskeletal rearrangements. The LIM-kinase 1 (LIMK-1) induces phosphorylation and inactivation of cofilin leading to increased actin polymerization (43). Cofilin-1 is involved in many cellular processes, including actin cytoskeletal remodeling and cellular metabolism (44, 45), and our group has found that loss of cofilin-1 contributes to the cytoskeletal remodeling that occurs with epithelial plasticity in response to chronic injury (32). Virus infection involves the remodeling of the actin cytoskeleton of the host cells (46, 47), and COVID-19 hijacks the cytoskeleton and causes barrier dysfunction (48, 49). In contrast, SCV2 infection in the nasal cells activates ROCK/LIMK signaling, leading to cofilin downregulation. Indeed, diminished inflammatory response in SCV2 infection than IAV has also suggested by the downregulation of cofilin as cofilin plays a key role in regulating the NLRP3 inflammasome (50). Previous studies have demonstrated that virus infections (transmissible gastroenteritis virus and porcine hemagglutinating encephalomyelitis) caused cofilin inactivation at the early stage of infection, leading to F-actin polymerization and rearrangement as well as further promoting virus entry (51, 52).

Given the involvement of cofilin-1, it was not surprising that SCV2 infection also caused dysregulated host metabolism compared to IAV infection, with decreased mitochondrial and mitochondrial-related gene expression, and increased glycolysis. Others have shown evidence that SCV2 reprograms host metabolism (53, 54). Both the ciliated epithelia and Type II alveolar epithelia are rich in mitochondria (55) with specialized activities (32, 56) and mitochondrial dysfunction could significantly impact their cellular function. This mitochondrial dysfunction is consistent with differential metabolomic profiles seen in patients with acute respiratory distress syndrome due to COVID-19 versus H1N1 influenza (57). The shift to aerobic glycolysis could favor SCV2 replication and survival through initial infection (58). We hypothesize that these findings represent strategies utilized by the cell to survive the infection but result in a fundamental shift in the epithelial phenotype, with potential long-term consequences, which could set the stage for the development of chronic lung disease or long COVID (59).

## MATERIALS AND METHODS

### Cell culture

Primary human nasal cells (hNECs) were obtained from patients with chronic rhinosinusitis (CRS) who tested negative for COVID-19 and were approved through Johns Hopkins Institutional Review. All subjects signed informed consent (60). Primary human bronchial epithelial cells (NHBE) were purchased from MatTek (Ashland, MA, USA) and cultured in PneumaCult medium (Stemcell Technology). The patient demographics were shown in Table S1. Briefly, the cells were cultured on the collagen I-coated transwell inserts with 0.4 µm pore (Corning) and differentiated for 4–6 weeks at the air-liquid interface (ALI) as described previously (32, 61). Human lung epithelial cells Calu3 were purchased from ATCC and cultured in EMEM (ATCC) with 10% fetal bovine serum (ThermoFisher Scientific) and 1% penicillin streptomycin (ThermoFisher Scientific). Calu3 cells were seeded onto the transwell with 0.4 µm pore at 5 × 10^4^ cells/cm^2^ and cultured at the ALI for 1 week.

### SARS-CoV-2 and influenza A infection in human nasal cells

Human nasal cells at ALI were infected with mock, SCV2 SARS-C0V-2/USA/HP7(27)/2020, and IAV A/Baltimore/R0243/2018 H3N2 at multiplicities of infection (MOI) of approximately 1.0 infectious unit per cell at the apical side for 120 min at 37°C followed by two PBS washes to eliminate unbound virus. The cells were incubated in a 37°C CO_2_ incubator for 12 or 24 hpi and then lysed with TRIZOL (ThermoFisher Scientific) for RNA isolation. Infectious virus titers were determined by median tissue culture infectious dose (TCID50), as described previously (62, 63).

### RNA sequencing and analysis

Three replicates were prepared for each infection (mock, IAV, SCV2) at 12 and 24 hpi. Extraction, purification, and quality assessment of Total RNA from hNECs were performed as previously described (64). Unique dual-index barcoded libraries for RNA-Seq were prepared from 125 ng total RNA using the Universal Plus Total RNA-Seq with NuQuant Library kit (Tecan Genomics), according to manufacturer’s recommended protocol, with amplification performed for 16 cycles, as optimized by qPCR. Quality assessment and quantitation of libraries were performed as previously described (64) including final quality assessment of the library pool on an Illumina iSeq Sequencer with iSeq100 i1 reagent V2 300 cycle kit. For deep RNA sequencing, a 200 cycle (2 × 100 bp) Illumina NovaSeq S1 run was performed at Johns Hopkins Genomics, Genetic Resources Core Facility, RRID:SCR_018669.

iSeq and NovaSeq data files were uploaded to the Partek Server, and analysis with Partek Flow NGS software, with RNA Toolkit, was performed as follows: pre-alignment QA/QC, trimming of reads, alignment to hg38 Reference Index using STAR 2.7.8 a, post-alignment QA/QC, quantification of gene counts to annotation model (Partek E/M, hg38-Ensembl Transcript Release 105), filter and normalization of gene counts, identification and comparison of differentially expressed genes with GSA (gene-specific analysis). From resulting gene lists, clustering and biological interpretation were performed. Hierarchical clustering was performed on gene lists SCV2 vs Mock, 12 and 24 h to generate heatmaps using Partek Flow defaults. The defaults include standardize for feature scaling and Euclidean and average linkage for sample and feature clustering. Tools from KEGG Mapper (https://www.genome.jp/kegg) were used for pathway analysis in the data set.

### Preparation of SCV2 pseudovirus and transduction

Full-length VSV glycoprotein (VSV-G) (Addgene, #12259) and pRP-Neo-EF1A-SARS-Cov2-Spike delta 18, fused with a DsRed tag, were kindly gifted from Vasudevan, Anand’s lab. Plasmid for the SARS-CoV-2 spike delta 18 glycoprotein has a C-terminal deletion of the last 18 amino acids to improve binding to ACE2. Human NHBE and Calu3 cells were infected with the pseudovirus with VSV-G or Spike-VSV-G at MOI 1 with 10 mg/mL of polybrene (Sigma-Aldrich) followed by centrifugation at 1500 rpm at room temperature for 2h. Then, the infection media were removed, and the cells were washed with PBS twice. The infected cells were incubated at 48 and 72 hpi for Calu3 and NHBE, respectively (>80% infection efficiency that was determined by DsRed fluorescent signal, data not shown). Cells were harvested at the designated time points.

For NHBE and Calu3 infected cells, at the desired time points, the cells were washed three times in phosphate-buffered saline (PBS) and then were fixed with 4% paraformaldehyde in PBS (Affymetrix Inc., OH, USA) for 15 min at room temperature. For human lungs, human lung was obtained from Johns Hopkins School of Medicine Pulmonary Department of Biorepository. Then, the lung tissues were embedded in paraffin and stained with hematoxylin and eosin (H&E) at Reference Histology Laboratory, Johns Hopkins Medical Institute–Pathology (Baltimore, USA). The patient demographics were shown in Table S1. The paraffin-embedded PCLS were deparaffinized in xylene followed by rehydration and antigen unmasking (Citrate Buffer pH:6.0).

### Immunofluorescence confocal microscopy

Both cells and lung tissues were subjected to immunofluorescence staining as described previously (65). Briefly, the slides were permeabilized with 0.1% Triton X-100 (Sigma-Aldrich) in PBS. Subsequently, they were blocked in 10% goat serum (Sigma-Aldrich) and 1% bovine serum albumin (Sigma-Aldrich) in 1× PBS for 1 h at room temperature. Next, they were incubated at 4°C overnight with primary antibodies rabbit against cofilin (D3F9) (1:200, Cell Signaling #5175), TOMM22 (1:200, Proteintech #11278-1-AP). After three washing with 1× PBS, the cells were incubated at the secondary antibody goat anti-rabbit IgG (H + L) cross-adsorbed secondary antibody, Alexa Fluor 555 (1:200, Invitrogen # A-21428) and Alexa Fluor 647 Phalloidin (1:400, Invitrogen #A22287) for 2 h at room temperature. The cells were stained with 1 µg/mL of Hoechst (ThermoFisher Scientific #62249) for visualization of the nuclei and mounted with ProLong Gold antifade (ThermoFisher Scientific). The slides were imaged using Zeiss LSM 700 Confocal or LSM880-AiryscanFAST with a 63 × oil objective. Images presented are a maximum projection of Z-stack images collected with LSM software unless the *xz*-plane was provided as with the tissue slices. Random fields were chosen for the imaging of cells, while all available sections with airways present were analyzed in the tissues.

### Reverse transcription-quantitative polymerase chain reaction

RNA was isolated using the NucleoSpin TriPrep kit (TaKaRa Bio USA, Inc.) according to the manufacturer’s instructions. One microgram of total RNA was reverse transcribed using an ABI High-capacity cDNA Reverse Transcription kit (Foster City, CA, USA). The complementary DNA was subjected to QPCR using Power SYBR Green Master Mix (ThermoFisher Scientific) and performed in StepOne Plus (ABI). The relative gene expression changes were normalized by housekeeping gene GAPDH and calculated using the 2−ΔΔCt method (66). The qPCR primers are listed in Table S2.

### Seahorse analysis

Cell Mitochondrial Stress (Agilent) was conducted to detect oxygen consumption rate and extracellular acidification rate. ATP production rate using Seahorse XFe24 Analyzer (Agilent) through the injection of 25 µg/mL oligomycin, 4 µM FCCP, 5 µM rotenone, and 10 µM antimycin A. Calu3 cells (1 × 10^5^ cells/cm^2^) were differentiated on the transwell with 0.4 µM pore for 1 week and infected with the pseudovirus. After 48 h infection, the cells were changed to Agilent Seahorse XF DMEM medium supplemented with 1 mM of sodium pyruvate, 2 mM of glutamine, and 1 mM of d-glucose and incubated at 37°C for 1 h. After washing with the assay buffer, OCR and ECAR were measured using Seahorse XF24 instrument and analyzed by Seahorse XF24 analyzer.

### Statistical analysis

Results were expressed as the mean with the error bars representing means (±standard error of the mean[SEM]). Statistics were determined by Welch’s test, with *P* < 0.05 considered statistically significant. All the data were analyzed and plotted by Prism 9.3 (Graphpad).

## Data Availability

All sequence files and sample information have been deposited at NCBI Sequence Read Archive, NCBI BioProject no. PRJNA952551.

## SUPPLEMENTAL MATERIAL

Tables S1 and S2 mbio.00820-23-s0001.pdf Table S1. Patient demographics of human lungs and NHBE cells. Table S2. Primer list.

ASM does not own the copyrights to Supplemental Material that may be linked to, or accessed through, an article. The authors have granted ASM a non-exclusive, world-wide license to publish the Supplemental Material files. Please contact the corresponding author directly for reuse.

## References

[1] Guan W-J , Ni Z-Y , Hu Y , Liang W-H , Ou C-Q , He J-X , Liu L , Shan H , Lei C-L , Hui DSC , Du B , Li L-J , Zeng G , Yuen K-Y , Chen R-C , Tang C-L , Wang T , Chen P-Y , Xiang J , Li S-Y , Wang J-L , Liang Z-J , Peng Y-X , Wei L , Liu Y , Hu Y-H , Peng P , Wang J-M , Liu J-Y , Chen Z , Li G , Zheng Z-J , Qiu S-Q , Luo J , Ye C-J , Zhu S-Y , Zhong N-S . 2020. Clinical characteristics of coronavirus disease 2019 in China. N Engl J Med 382:1708–1720. doi:10.1056/NEJMoa2002032 32109013 PMC7092819

[2] Huang C , Wang Y , Li X , Ren L , Zhao J , Hu Y , Zhang L , Fan G , Xu J , Gu X , Cheng Z , Yu T , Xia J , Wei Y , Wu W , Xie X , Yin W , Li H , Liu M , Xiao Y , Gao H , Guo L , Xie J , Wang G , Jiang R , Gao Z , Jin Q , Wang J , Cao B . 2020. Clinical features of patients infected with 2019 novel coronavirus in Wuhan, China. Lancet 395:497–506. doi:10.1016/S0140-6736(20)30183-5 31986264 PMC7159299

[3] Hoffmann M , Kleine-Weber H , Schroeder S , Krüger N , Herrler T , Erichsen S , Schiergens TS , Herrler G , Wu NH , Nitsche A , Müller MA , Drosten C , Pöhlmann S . 2020. SARS-CoV-2 cell entry depends on ACE2 and TMPRSS2 and is blocked by a clinically proven protease inhibitor. Cell 181:271–280. doi:10.1016/j.cell.2020.02.052 32142651 PMC7102627

[4] Letko M , Marzi A , Munster V . 2020. Functional assessment of cell entry and receptor usage for SARS-CoV-2 and other lineage B betacoronaviruses. Nat Microbiol 5:562–569. doi:10.1038/s41564-020-0688-y 32094589 PMC7095430

[5] Dou D , Revol R , Östbye H , Wang H , Daniels R . 2018. Influenza A virus cell entry, replication, virion assembly and movement. Front Immunol 9:1581. doi:10.3389/fimmu.2018.01581 30079062 PMC6062596

[6] Sakai T , Nishimura SI , Naito T , Saito M . 2017. Influenza A virus hemagglutinin and neuraminidase act as novel motile machinery. Sci Rep 7:45043. doi:10.1038/srep45043 28344335 PMC5366856

[7] Johns Hopkins Medicine . 2022. COVID-19 vs. the flu. Available from: https://www.hopkinsmedicine.org/health/conditions-and-diseases/coronavirus/coronavirus-disease-2019-vs-the-flu

[8] Han X , Chen L , Fan Y , Alwalid O , Jia X , Zheng Y , Liu J , Li Y , Cao Y , Gu J , Liu J , Zheng C , Ye Q , Shi H . 2023. Longitudinal assessment of chest CT findings and pulmonary function after COVID-19 infection. Radiology 307:e222888. doi:10.1148/radiol.222888 36786698 PMC9969419

[9] Desai AD , Lavelle M , Boursiquot BC , Wan EY . 2022. Long-term complications of COVID-19. Am J Physiol Cell Physiol 322:C1–C11. doi:10.1152/ajpcell.00375.2021 34817268 PMC8721906

[10] Gao KM , Derr AG , Guo Z , Nündel K , Marshak-Rothstein A , Finberg RW , Wang JP . 2021. Human nasal wash RNA-Seq reveals distinct cell-specific innate immune responses in influenza versus SARS-CoV-2. JCI Insight 6:e152288. doi:10.1172/jci.insight.152288 34618691 PMC8663782

[11] Flerlage T , Boyd DF , Meliopoulos V , Thomas PG , Schultz-Cherry S . 2021. Influenza virus and SARS-CoV-2: pathogenesis and host responses in the respiratory tract. Nat Rev Microbiol 19:425–441. doi:10.1038/s41579-021-00542-7 33824495 PMC8023351

[12] Donnino MW , Moskowitz A , Thompson GS , Heydrick SJ , Pawar RD , Berg KM , Mehta S , Patel PV , Grossestreuer AV . 2021. Comparison between patients hospitalized with influenza and COVID-19 at a tertiary care center. J Gen Intern Med 36:1689–1695. doi:10.1007/s11606-021-06647-2 33738759 PMC7971402

[13] Vareille M , Kieninger E , Edwards MR , Regamey N . 2011. The airway epithelium: soldier in the fight against respiratory viruses. Clin Microbiol Rev 24:210–229. doi:10.1128/CMR.00014-10 21233513 PMC3021210

[14] Bridges JP , Vladar EK , Huang H , Mason RJ . 2022. Respiratory epithelial cell responses to SARS-CoV-2 in COVID-19. Thorax 77:203–209. doi:10.1136/thoraxjnl-2021-217561 34404754 PMC9273148

[15] Sungnak W , Huang N , Bécavin C , Berg M , Queen R , Litvinukova M , Talavera-López C , Maatz H , Reichart D , Sampaziotis F , Worlock KB , Yoshida M , Barnes JL , Banovich NE , Barbry P , Brazma A , Collin J , Desai TJ , Duong TE , Eickelberg O , Falk C , Farzan M , Glass I , Gupta RK , Haniffa M , Horvath P , Hubner N , Hung D , Kaminski N , Krasnow M , Kropski JA , Kuhnemund M , Lako M , Lee H , Leroy S , Linnarson S , Lundeberg J , Meyer KB , Miao Z , v. MA , Nawijn MC , Nikolic MZ , Noseda M , Ordovas-Montanes J , Oudit GY , Pe’er D , Powell J , Quake S , Rajagopal J , Tata PR , Rawlins EL , Regev A , Reyfman PA , Rozenblatt-Rosen O , Saeb-Parsy K , Samakovlis C , Schiller HB , Schultze JL , Seibold MA , Seidman CE , Seidman JG , Shalek AK , Shepherd D , Spence J , Spira A , Sun X , Teichmann SA , Theis FJ , Tsankov AM , Vallier L , Berge M , Whitsett J , Xavier R , Xu Y , Zaragosi LE , Zerti D , Zhang H , Zhang K , Rojas M , Figueiredo F . 2020. SARS-CoV-2 entry factors are highly expressed in nasal epithelial cells together with innate immune genes. Nat Med 26:681–687. doi:10.1038/s41591-020-0868-6 32327758 PMC8637938

[16] Hou YJ , Okuda K , Edwards CE , Martinez DR , Asakura T , Dinnon KH III , Kato T , Lee RE , Yount BL , Mascenik TM , Chen G , Olivier KN , Ghio A , Tse LV , Leist SR , Gralinski LE , Schäfer A , Dang H , Gilmore R , Nakano S , Sun L , Fulcher ML , Livraghi-Butrico A , Nicely NI , Cameron M , Cameron C , Kelvin DJ , de Silva A , Margolis DM , Markmann A , Bartelt L , Zumwalt R , Martinez FJ , Salvatore SP , Borczuk A , Tata PR , Sontake V , Kimple A , Jaspers I , O’Neal WK , Randell SH , Boucher RC , Baric RS . 2020. SARS-CoV-2 reverse genetics reveals a variable infection gradient in the respiratory tract. Cell 182:429–446. doi:10.1016/j.cell.2020.05.042 32526206 PMC7250779

[17] Ziegler CGK , Allon SJ , Nyquist SK , Mbano IM , Miao VN , Tzouanas CN , Cao Y , Yousif AS , Bals J , Hauser BM , Feldman J , Muus C , Wadsworth MH II , Kazer SW , Hughes TK , Doran B , Gatter GJ , Vukovic M , Taliaferro F , Mead BE , Guo Z , Wang JP , Gras D , Plaisant M , Ansari M , Angelidis I , Adler H , Sucre JMS , Taylor CJ , Lin B , Waghray A , Mitsialis V , Dwyer DF , Buchheit KM , Boyce JA , Barrett NA , Laidlaw TM , Carroll SL , Colonna L , Tkachev V , Peterson CW , Yu A , Zheng HB , Gideon HP , Winchell CG , Lin PL , Bingle CD , Snapper SB , Kropski JA , Theis FJ , Schiller HB , Zaragosi L-E , Barbry P , Leslie A , Kiem H-P , Flynn JL , Fortune SM , Berger B , Finberg RW , Kean LS , Garber M , Schmidt AG , Lingwood D , Shalek AK , Ordovas-Montanes J , Banovich N , Barbry P , Brazma A , Desai T , Duong TE , Eickelberg O , Falk C , Farzan M , Glass I , Haniffa M , Horvath P , Hung D , Kaminski N , Krasnow M , Kropski JA , Kuhnemund M , Lafyatis R , Lee H , Leroy S , Linnarson S , Lundeberg J , Meyer K , Misharin A , Nawijn M , Nikolic MZ , Ordovas-Montanes J , Pe’er D , Powell J , Quake S , Rajagopal J , Tata PR , Rawlins EL , Regev A , Reyfman PA , Rojas M , Rosen O , Saeb-Parsy K , Samakovlis C , Schiller H , Schultze JL , Seibold MA , Shalek AK , Shepherd D , Spence J , Spira A , Sun X , Teichmann S , Theis F , Tsankov A , van den Berge M , von Papen M , Whitsett J , Xavier R , Xu Y , Zaragosi L-E , Zhang K . 2020. SARS-CoV-2 receptor ACE2 is an interferon-stimulated gene in human airway epithelial cells and is detected in specific cell subsets across tissues. Cell 181:1016–1035. doi:10.1016/j.cell.2020.04.035 32413319 PMC7252096

[18] Ghosh B , Park B , Bhowmik D , Nishida K , Lauver M , Putcha N , Gao P , Ramanathan M , Hansel N , Biswal S , Sidhaye VK . 2020. RAPID REPORT translational physiology strong correlation between air-liquid interface cultures and in vivo transcriptomics of nasal brush biopsy. Am J Physiol Lung Cell Mol Physiol 318:L1056–L1062. doi:10.1152/ajplung.00050.2020 32233789 PMC7272738

[19] Mudd PA , Crawford JC , Turner JS , Souquette A , Reynolds D , Bender D , Bosanquet JP , Anand NJ , Striker DA , Martin RS , Boon ACM , House SL , Remy KE , Hotchkiss RS , Presti RM , O’Halloran JA , Powderly WG , Thomas PG , Ellebedy AH . 2020. Distinct inflammatory profiles distinguish COVID-19 from influenza with limited contributionsfrom cytokine storm. Sci Adv 6. doi:10.1126/sciadv.abe3024 PMC7725462 33187979

[20] Rodríguez LL . 2002. Emergence and re-emergence of vesicular stomatitis in the United States virus research. Virus Res 85:211–219. doi:10.1016/s0168-1702(02)00026-6 12034487

[21] Lord CC , Tabachnick WJ . 2002. Influence of nonsystemic transmission on the epidemiology of insect borne Arboviruses: a case study of vesicular stomatitis epidemiology in the Western United States. J Med Entomol 39:417–426. doi:10.1603/0022-2585-39.3.417 12061433

[22] Zhang J , Xiang H , Liu J , Chen Y , He RR , Liu B . 2020. Mitochondrial Sirtuin 3: new emerging biological function and therapeutic target. Theranostics 10:8315–8342. doi:10.7150/thno.45922 32724473 PMC7381741

[23] Chu H , Chan J-W , Yuen T-T , Shuai H , Yuan S , Wang Y , Hu B , Yip C-Y , Tsang J-L , Huang X , Chai Y , Yang D , Hou Y , Chik K-H , Zhang X , Fung A-F , Tsoi H-W , Cai J-P , Chan W-M , Ip JD , Chu A-H , Zhou J , Lung DC , Kok K-H , To K-W , Tsang O-Y , Chan K-H , Yuen K-Y . 2020. Comparative tropism, replication kinetics, and cell damage profiling of SARS-CoV-2 and SARS-CoV with implications for clinical manifestations, transmissibility, and laboratory studies of COVID-19: an observational study. Lancet Microbe 1:e14–e23. doi:10.1016/S2666-5247(20)30004-5 32835326 PMC7173822

[24] Harcourt JL , Haynes LM . 2013. Establishing a liquid-covered culture of polarized human airway epithelial Calu-3 cells to study host cell response to respiratory pathogens in Vitro. J Vis Exp:50157. doi:10.3791/50157 23426201 PMC3600762

[25] Min KA , Rosania GR , Kim C-K , Shin MC . 2016. Functional and cytometric examination of different human lung epithelial cell types as drug transport barriers. Arch Pharm Res 39:359–369. doi:10.1007/s12272-015-0704-6 26746641 PMC4794378

[26] Cagno V . 2020. SARS-CoV-2 cellular tropism. Lancet Microbe 1:e2–e3. doi:10.1016/S2666-5247(20)30008-2 32835319 PMC7173832

[27] Florea BI , Cassara ML , Junginger HE , Borchard G . 2003. Drug transport and metabolism characteristics of the human airway epithelial cell line Calu-3. J Control Release 87:131–138. doi:10.1016/s0168-3659(02)00356-5 12618029

[28] Pascoe CD , Roy N , Turner-Brannen E , Schultz A , Vaghasiya J , Ravandi A , Halayko AJ , West AR . 2021. Oxidized phosphatidylcholines induce multiple functional defects in airway epithelial cells. Am J Physiol Lung Cell Mol Physiol 321:L703–L717. doi:10.1152/ajplung.00539.2020 34346781

[29] Petcherski A , Sharma M , Satta S , Daskou M , Vasilopoulos H , Hugo C , Ritou E , Dillon BJ , Fung E , Garcia G , Scafoglio C , Purkayastha A , Gomperts BN , Fishbein GA , Arumugaswami V , Liesa M , Shirihai OS , Kelesidis T . 2022. Mitoquinone mesylate targets SARS-CoV-2 infection in preclinical models. bioRxiv:2022.02.22.481100. doi:10.1101/2022.02.22.481100

[30] Rezaee F , Georas SN . 2014. Breaking barriers: new insights into airway epithelial barrier function in health and disease. Am J Respir Cell Mol Biol 50:857–869. doi:10.1165/rcmb.2013-0541RT 24467704 PMC4068951

[31] Fletcher DA , Mullins RD . 2010. Cell mechanics and the cytoskeleton. Nature 463:485–492. doi:10.1038/nature08908 20110992 PMC2851742

[32] Ghosh B , Nishida K , Chandrala L , Mahmud S , Thapa S , Swaby C , Chen S , Khosla AA , Katz J , Sidhaye VK . 2022. Epithelial plasticity in COPD results in cellular unjamming due to an increase in polymerized actin. J Cell Sci 135:jcs258513. doi:10.1242/jcs.258513 35118497 PMC8919336

[33] Lui W , Lee WM , Cheng CY . 2003. Sertoli-germ cell adherens junction dynamics in the testis are regulated by RhoB GTPase via the ROCK/LIMK signaling pathway. Biol Reprod 68:2189–2206. doi:10.1095/biolreprod.102.011379 12606349

[34] Koutsakos M , McWilliam HEG , Aktepe TE , Fritzlar S , Illing PT , Mifsud NA , Purcell AW , Rockman S , Reading PC , Vivian JP , Rossjohn J , Brooks AG , Mackenzie JM , Mintern JD , Villadangos JA , Nguyen THO , Kedzierska K . 2019. Downregulation of MHC class I expression by influenza A and B viruses. Front Immunol 10:1158. doi:10.3389/fimmu.2019.01158 31191533 PMC6548845

[35] Papi A , Stanciu LA , Papadopoulos NG , Teran LM , Holgate ST , Johnston SL . 2000. Rhinovirus infection induces major histocompatibility complex class I and costimulatory molecule upregulation on respiratory epithelial cells. J Infect Dis 181:1780–1784. doi:10.1086/315463 10823784

[36] Goel S , Saheb Sharif-Askari F , Saheb Sharif Askari N , Madkhana B , Alwaa AM , Mahboub B , Zakeri AM , Ratemi E , Hamoudi R , Hamid Q , Halwani R . 2021. SARS-CoV-2 switches ‘on’ MAPK and NFκB signaling via the reduction of nuclear DUSP1 Anddusp5 expression. Front Pharmacol 12:631879. doi:10.3389/fphar.2021.631879 33995033 PMC8114414

[37] Liao Y , Wang X , Huang M , Tam JP , Liu DX . 2011. Regulation of the p38 mitogen-activated protein kinase and dual-specificity phosphatase 1 feedback loop modulates the induction of interleukin 6 and 8 in cells infected with coronavirus infectious bronchitis virus. Virology 420:106–116. doi:10.1016/j.virol.2011.09.003 21959016 PMC7111953

[38] Blanco-Melo D , Nilsson-Payant BE , Liu W-C , Møller R , Panis M , Sachs D , Albrecht RA , tenOever BR . 2020. SARS-CoV-2 launches a unique transcriptional signature from in vitro, ex vivo, and in vivo systems. bioRxiv. doi:10.1101/2020.03.24.004655

[39] Collins BJ , Kleeberger W , Ball DW . 2004. Notch in lung development and lung cancer. Semin Cancer Biol 14:357–364. doi:10.1016/j.semcancer.2004.04.015 15288261

[40] Baindara P , Sarker MB , Earhart AP , Mandal SM , Schrum AG . 2022. NOTCH signaling in COVID-19: a central hub controlling genes, proteins, and cells that mediate SARS-CoV-2 entry, the inflammatory response, and lung regeneration. Front Cell Infect Microbiol 12:928704. doi:10.3389/fcimb.2022.928704 35992174 PMC9386183

[41] Turpaev KT . 2020. Transcription factor KLF2 and its role in the regulation of inflammatory processes. Biochemistry (Mosc) 85:54–67. doi:10.1134/S0006297920010058 32079517

[42] Wu D , Lee T-H , Huang R-T , D Guzy R , Schoettler N , Adegunsoye A , Mueller J , Husain A , I Sperling A , Mutlu GM , Fang Y . 2021. SARS-CoV-2 infection is associated with reduced Krüppel-like factor 2 in human lung autopsy. Am J Respir Cell Mol Biol 65:222–226. doi:10.1165/rcmb.2020-0564LE 33971111 PMC8399572

[43] Yang N , Higuchi O , Ohashi K , Nagata K , Wada A , Kangawa K , Nishida E , Mizuno K . 1998. Cofilin Phosphorylation by LIM-kinase 1 and its role in Rac-mediated actin reorganization. Nature 393:809–812. doi:10.1038/31735 9655398

[44] Hoffmann L , Waclawczyk MS , Tang S , Hanschmann E-M , Gellert M , Rust MB , Culmsee C . 2021. Cofilin1 oxidation links oxidative distress to mitochondrial demise and neuronal cell death. Cell Death Dis 12:953. doi:10.1038/s41419-021-04242-1 34657120 PMC8520533

[45] Rehklau K , Hoffmann L , Gurniak CB , Ott M , Witke W , Scorrano L , Culmsee C , Rust MB . 2017. Cofilin1-dependent actin dynamics control DRP1-mediated mitochondrial fission. Cell Death Dis 8:e3063. doi:10.1038/cddis.2017.448 28981113 PMC5680571

[46] Dohner K , Sodeik B. 2005. The Role of the cytoskeleton during viral infection, p. 67–108. In Current Topics in Microbiology and Immunology . 10.1007/3-540-26764-6_3 15609501

[47] Taylor MP , Koyuncu OO , Enquist LW . 2011. Subversion of the actin cytoskeleton during viral infection. Nat Rev Microbiol 9:427–439. doi:10.1038/nrmicro2574 21522191 PMC3229036

[48] Aminpour M , Hameroff S , Tuszynski JA . 2022. How COVID-19 Hijacks the cytoskeleton: therapeutic implications. Life (Basel) 12:814. doi:10.3390/life12060814 35743845 PMC9225596

[49] Deinhardt-Emmer S , Böttcher S , Häring C , Giebeler L , Henke A , Zell R , Jungwirth J , Jordan PM , Werz O , Hornung F , Brandt C , Marquet M , Mosig AS , Pletz MW , Schacke M , Rödel J , Heller R , Nietzsche S , Löffler B , Ehrhardt C . 2021. SARS-CoV-2 causes severe epithelial inflammation and barrier dysfunction. J Virol 95:e00110-21. doi:10.1128/JVI.00110-21 33637603 PMC8139673

[50] Park Y , Kastner D , Chae JJ . 2015. Cofilin-1 is an essential redox sensor for NLRP3 Inflammasome activation. Pediatr Rheumatol 13. doi:10.1186/1546-0096-13-S1-O52

[51] Hu W , Zhu L , Yang X , Lin J , Yang Q . 2016. The epidermal growth factor receptor regulates cofilin activity and promotes transmissible gastroenteritis virus entry into intestinal epithelial cells. Oncotarget 7:12206–12221. doi:10.18632/oncotarget.7723 26933809 PMC4914279

[52] Lv X , Li Z , Guan J , Hu S , Zhang J , Lan Y , Zhao K , Lu H , Song D , He H , Gao F , He W . 2019. Porcine hemagglutinating encephalomyelitis virus activation of the integrin Α5Β1-FAK-Cofilin pathway causes cytoskeletal rearrangement to promote its invasion of N2A cells. J Virol 93:e01736-18. doi:10.1128/JVI.01736-18 30541856 PMC6384086

[53] Moolamalla STR , Balasubramanian R , Chauhan R , Priyakumar UD , Vinod PK . 2021. Host metabolic Reprogramming in response to SARS-CoV-2 infection: a systems biology approach. Microb Pathog 158:105114. doi:10.1016/j.micpath.2021.105114 34333072 PMC8321700

[54] Zhang Y , Guo R , Kim SH , Shah H , Zhang S , Liang JH , Fang Y , Gentili M , Leary CNO , Elledge SJ , Hung DT , Mootha VK , Gewurz BE . 2021. SARS-CoV-2 Hijacks folate and one-carbon metabolism for viral replication. Nat Commun 12. doi:10.1038/s41467-021-21903-z PMC7960988 33723254

[55] Cloonan SM , Kim K , Esteves P , Trian T , Barnes PJ . 2020. Mitochondrial dysfunction in lung ageing and disease. Eur Respir Rev 29:200165. doi:10.1183/16000617.0165-2020 33060165 PMC9488551

[56] Kliment CR , Nguyen JMK , Kaltreider MJ , Lu Y , Claypool SM , Radder JE , Sciurba FC , Zhang Y , Gregory AD , Iglesias PA , Sidhaye VK , Robinson DN . 2021. Adenine nucleotide translocase regulates airway epithelial metabolism, surface hydration and ciliary function. J Cell Sci 134:jcs257162. doi:10.1242/jcs.257162 33526710 PMC7929926

[57] Lorente JA , Nin N , Villa P , Vasco D , Miguel-Coello AB , Rodriguez I , Herrero R , Peñuelas O , Ruiz-Cabello J , Izquierdo-Garcia JL . 2021. Metabolomic diferences between COVID-19 and H1N1 influenza induced ARDS. Crit Care 25. doi:10.1186/s13054-021-03810-3 PMC8591432 34781986

[58] Icard P , Lincet H , Wu Z , Coquerel A , Forgez P , Alifano M , Fournel L . 2021. The key role of Warburg effect in SARS-CoV-2 replication and associated inflammatory response. Biochimie 180:169–177. doi:10.1016/j.biochi.2020.11.010 33189832 PMC7659517

[59] Smirnova L , Harris G , Leist M , Hartung T . 2015. Food for thought⋯cellular resilience. ALTEX 32:247–260. doi:10.14573/altex.1509271 26536287

[60] Tharakan A , Halderman AA , Lane AP , Biswal S , Ramanathan M . 2016. Reversal of cigarette smoke extract-induced sinonasal epithelial cell barrier dysfunction through Nrf2 activation. Int Forum Allergy Rhinol 6:1145–1150. doi:10.1002/alr.21827 27580429 PMC5097009

[61] Ramanathan M , Spannhake EW , Lane AP . 2007. Chronic Rhinosinusitis with nasal polyps is associated with decreased expression of Mucosal interleukin 22 receptor. Laryngoscope 117:1839–1843. doi:10.1097/MLG.0b013e31811edd4f 17906500

[62] Klein SL , Pekosz A , Park H-S , Ursin RL , Shapiro JR , Benner SE , Littlefield K , Kumar S , Naik HM , Betenbaugh MJ , Shrestha R , Wu AA , Hughes RM , Burgess I , Caturegli P , Laeyendecker O , Quinn TC , Sullivan D , Shoham S , Redd AD , Bloch EM , Casadevall A , Tobian AAR . 2020. Sex, age, and hospitalization drive antibody responses in a COVID-19 convalescent plasma donor population. J Clin Invest 130:6141–6150. doi:10.1172/JCI142004 32764200 PMC7598041

[63] Wohlgemuth N , Lane AP , Pekosz A . 2018. Influenza A virus M2 protein apical targeting is required for efficient virus replication. J Virol 92:e01425-18. doi:10.1128/JVI.01425-18 30158290 PMC6206479

[64] Resnick JD , Beer MA , Pekosz A . 2023. Early transcriptional responses of human nasal epithelial cells to infection with Influenza A and SARS-CoV-2 virus differ and are influenced by physiological temperature. Pathogens 12:480. doi:10.3390/pathogens12030480 36986402 PMC10051809

[65] Ghosh B , Loube J , Thapa S , Ryan H , Capodanno E , Chen D , Swaby C , Chen S , Mahmud S , Girgis M , Nishida K , Ying L , Chengala PP , Tieng E , Burnim M , Wally A , Bhowmik D , Zaykaner M , Yeung-Luk B , Mitzner W , Biswal S , Sidhaye VK . 2022. Loss of E-cadherin is causal to pathologic changes in chronic lung disease. Commun Biol 5:1149. doi:10.1038/s42003-022-04150-w 36309587 PMC9617938

[66] Livak KJ , Schmittgen TD . 2001. Analysis of relative gene expression data using real-time quantitative PCR and the 2-ΔΔCT method. Methods 25:402–408. doi:10.1006/meth.2001.1262 11846609

