# Supplementary material for: SARS-CoV-2 infection alters mitochondrial and cytoskeletal function in human respiratory epithelial cells mediated by expression of spike protein
Source: mBio. 2023 Jul 28;14(4):e00820-23. doi: 10.1128/mbio.00820-23 (PMC10470579; doi:10.1128/mbio.00820-23)
Supplement: Tables S1 and S2 — Table S1. Patient demographics of human lungs and NHBE cells. Table S2. Primer list. [file mbio.00820-23-s0001.pdf]

TABLE S1. Patient demographics of human lungs and NHBE cells.

Human lungs					
**ID**	**Age**	**Sex**	**Race**	**Known tobacco history**	**COVID-19 Pneumonia**
Human lungs					
Normal lung	55	F	White or caucasian	Never smoker	No
COVID lung 1	79	F	Asian	Former smoker	Yes
COVID lung 2	66	F	Black or African American	Never smoker	Yes
NHBE					
NHBE 1	59	Female	Caucasian	Non-smoker	
NHBE 2	52	Male	Caucasian	Non-smoker	
NHBE 3	52	Female	Hispanic	Non-smoker	

TABLE S2. Primer list.

Gene	Forward primer (5' to 3')	Reverse primer (5' to 3')
CFL1	GGTGCTCTTCTGCCTGAGTG	TCTTGACAAAGGTGGCGTAG
HES1	ACGACACCGGATAAACCAAAGA	ATGCCGCGAGCTATCTTTCT
GAPDH	AACGGGAAGCTCACTGGCATG	TCCACCACCTGTTGCTGTAG
KFL2	CACGCACACAGGTGAGAAG	CCGTGTGCTTTCGGTAGTGG
PKLR	AAGAAGTAGGCTGGCACGACGT	GCGGATGTTCTCCACAATGGAC
RHOB	GGTCCCCTGAGCATGCTTTT	GAGGGGAGTCGAACAGACAC
SIRT3	GAGCTTCTGGGCTGGACAGA	TGGGATGTGGATGTCTCCTATG
TOMM22	AATTGCTCCCGAAAGGCGA	GACAGGGTCTCATCTAGCTCC
